# Uses of infrared thermography in acute illness: a systematic review

**DOI:** 10.3389/fmed.2024.1412854

**Published:** 2024-06-24

**Authors:** Sophie A. Stanley, Pip Divall, Jonathan P. Thompson, Matthew Charlton

**Affiliations:** ^1^Lancaster Medical School, University of Lancaster, Lancaster, United Kingdom; ^2^Department of Anaesthesia, The Royal Oldham Hospital, Oldham, United Kingdom; ^3^University Hospitals of Leicester NHS Trust, Leicester, United Kingdom; ^4^Department of Cardiovascular Sciences, University of Leicester, Leicester, United Kingdom

**Keywords:** infrared thermography, critical care, emergency medicine, paediatric intensive care (PICU), infrared thermal (IRT) imager

## Abstract

**Introduction:**

Infrared thermography (IRT) is a non-contact, non-ionising imaging modality, providing a visual representation of temperature distribution across a surface.

**Methods:**

We conducted a systematic search of indexed and grey literature for studies investigating IRT applications involving patients in acute care settings. Studies were categorised and described along themes identified iteratively using narrative synthesis. Quality appraisal of included studies was performed using the Quality Assessment tool for Diagnostic Accuracy Studies.

**Results:**

Of 1,060 unique records, 30 studies were included. These were conducted in emergency departments and intensive care units involving adult, paediatric and neonatal patients. IRT was studied for the diagnosis, monitoring or risk stratification of a wide range of individual conditions. IRT was predominantly used to display thermal change associated with localised inflammation or microcirculatory dysfunction. Existing research is largely at an early developmental stage.

**Discussion:**

We recommend that high quality diagnostic validation studies are now required for some clinical applications. IRT has the potential to be a valuable tool in the acute care setting and represents an important area for future research particularly when combined with advances in machine learning technology.

**Systematic review registration:**

CRD 42022327619 (https://www.crd.york.ac.uk/prospero/display_record.php?RecordID=327619).

## 1 Introduction

Infrared thermography (IRT) is a non-contact, affordable imaging modality that is easy to use. It can provide visual representations of the distribution of temperatures across a surface and gained interest for the mass detection of fever in individuals during the COVID-19 pandemic (1). Interest in IRT was similarly observed following the severe acute respiratory syndrome (SARS) epidemic in 2003, which stimulated technological advancements and the development of international standards to guide its use (1, 2). This inspired many new potential applications for IRT. Since the 1990’s, the elimination of thermistor cooling requirements has reduced the size and increased the portability of IRT devices (3). Performance has also been improved through larger capacity viewing fields, improved spatial and temperature resolutions and increased temperature sensitivity (3). Consumer interest in using IRT in home security systems and vehicles has improved the affordability of commercial devices (3).

These advancements have led to the exploration and development of IRT for a wide range of medical applications (4). Previous research has demonstrated that IRT can provide novel supplementary diagnostic information, for example in the management of inflammatory joint disease or breast cancer (3, 4). The inherent features of IRT (it is non-contact, non-invasive, provides real-time information and uses non-ionising radiation) have been recognised as unique advantages compared with other imaging technologies.

Research into the applications of IRT in acute care is expanding although has never previously been systematically reviewed. Therefore, we undertook a systematic review to describe and classify the acute care applications for IRT. The evidence for these applications is also critically appraised to indicate areas for future development.

## 2 Method

A systematic review was undertaken following the Preferred Reporting Items for Systematic Reviews and Meta Analyses (PRISMA) guidelines (5). The review protocol was registered on PROSPERO (CRD42022327619).

### 2.1 Inclusion criteria

We included all study types that applied IRT for any purpose to patients in an acute care setting. A broad definition of acute care was used to include all locations where critically unwell patients may be cared for. This included intensive care units, emergency departments, acute medical units, or the prehospital setting. Participants could be human patients of any age with any underlying pathology.

We excluded opinion pieces, preprints, conference abstracts and animal studies. Studies where IRT was used as a tool in the assessment of something else (rather than being the subject of the investigation), studies in simulated models or healthy volunteers only, and studies where IRT was used to study the acute care environment, staff or equipment (without reference to use on patients) were also excluded. Studies investigating only infrared thermometry or only thermal imaging were excluded. Studies could only be included if the technique being studied was combined infrared thermal imaging. Studies containing fewer than 10 participants were excluded.

### 2.2 Database searches

Electronic databases of published literature (Medline, Embase, Web of Science, CINAHL and ISRCTN registry) were searched on 27 March 2024. MedRxiv was searched on 27 March 2024 to identify grey literature.

A search strategy was developed with a specialist clinical librarian (PD) in Medline from Medical Subject Headings and free text terms then adapted for other databases. The detailed search strategies are available in the Supplementary material. There were no publication date or language restrictions applied to the search.

### 2.3 Screening

Search results were merged in Refworks (Clarivate Analytics) and deduplicated. Titles and abstracts were independently double screened in Rayyan systematic review software (6) according to the prespecified inclusion/ exclusion criteria by two authors (SAS, MC). Full-text articles for potential inclusion were retrieved and independently double screened for eligibility by two authors (SAS, MC). The final list of included studies was agreed by four authors (SAS, MC, JT, PD). In cases of disagreement or uncertainty, conflicts were resolved by discussion between screening authors with the option to consult a third independent reviewer if required.

### 2.4 Data extraction

A spreadsheet template in Microsoft Excel (Version 16.83) was developed in accordance with the Cochrane Handbook (7) for data extraction by a single author (SAS). Extracted data were verified by a second author (MC). Final data was agreed by all four authors (SAS, MC, JT, PD). Original authors of eligible papers were contacted by email for additional information if required.

We extracted the following publication data (publication title, authors, date of publication, journal, funding source, conflicts of interest, lead author’s contact details); methodological data (study design, ethical approval, population description, eligibility criteria, recruitment method, consent, randomisation method if applicable); setting data (geographical and clinical location); participant data (number of participants, age range, underlying pathology, specific condition of interest, difference between groups if randomised); intervention (IRT) data (advantages of IRT referenced, theory behind IRT application, variables measured using IRT, limitations of IRT described); reference standard data (variables measured, technique used); and outcome data (person reporting IRT and reference outcomes, missing data, power/sample size calculation, study findings).

### 2.5 Data synthesis

The heterogeneity of the study types and applications suggested by scoping searches meant that a narrative synthesis and qualitative appraisal was undertaken iteratively. This comprised four main elements: theory development, preliminary synthesis, exploring relationships and assessing the robustness of the synthesis (8). Studies were grouped initially according to application of the technology (condition or purpose or location). The quality and relationship between studies within each grouping was explored using conceptual and methodological triangulation. Themes were identified inductively through this process. The final themes were discussed and agreed by all authors (SAS, MC, JT, PD).

### 2.6 Quality appraisal

The studies were appraised using the Quality Assessment tool for Diagnostic Accuracy Studies (QUADAS-2) by two authors (SAS, MC) as scoping searches suggested that most applications of IRT would be diagnostic (9). For non-diagnostic studies, the QUADAS-2 tool was adapted with reference to the Critical Appraisal Skills Programme (CASP) checklist specific to that study type (10).

## 3 Results

**Figure 1:**
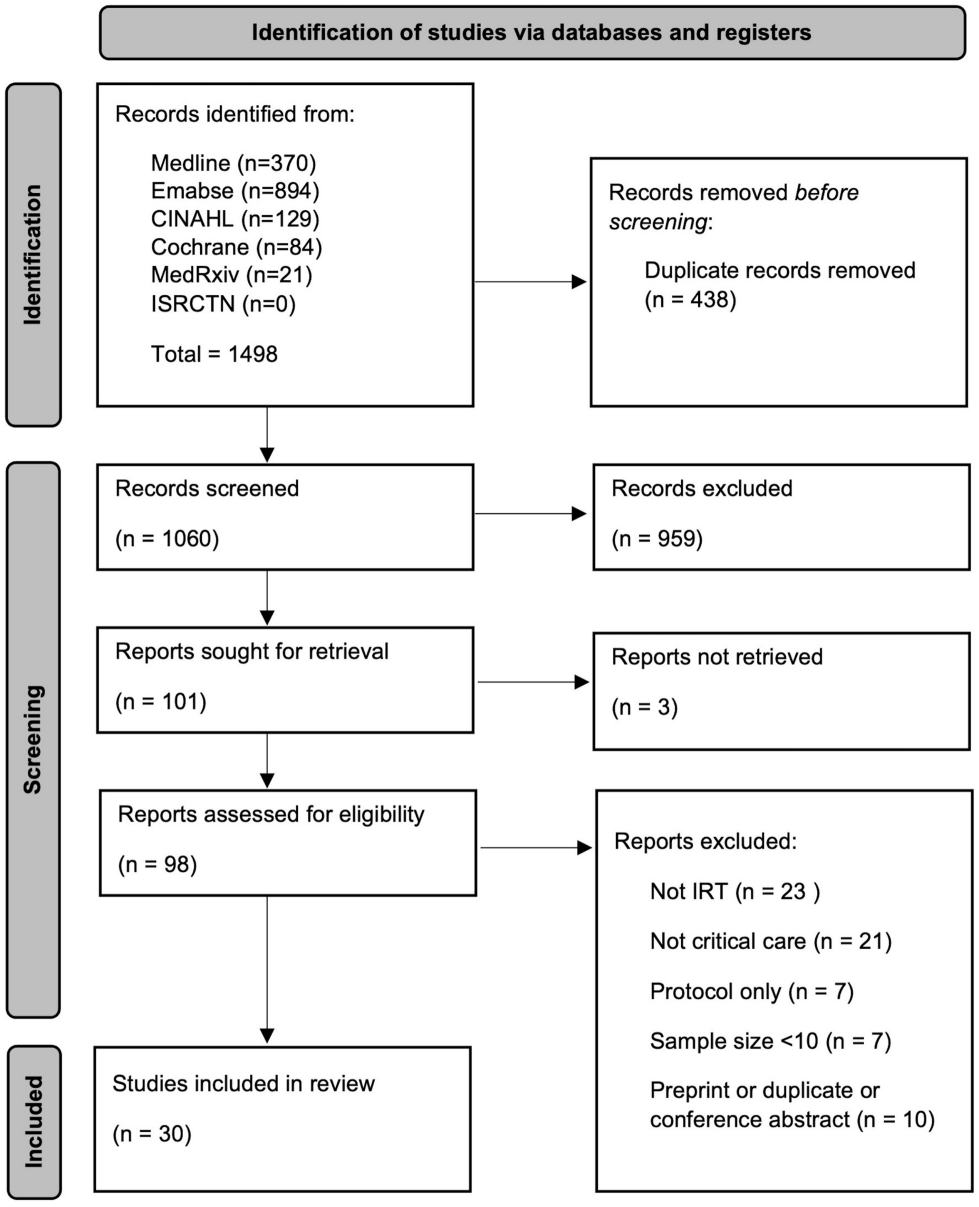
Preferred Reporting Items for Systematic Reviews and Meta-Analyses (PRISMA) flow diagram (5), CINAHL, cumulative index to nursing and allied health literature database; ISRCTN, international standard randomised controlled trial number registry; IRT, infrared thermography.

Searches identified 1,060 records after deduplication. Title screening excluded 959 records. Three full texts could not be retrieved, therefore 98 records were assessed for eligibility, of which 30 were included for review (Figure 1). The characteristics of the included studies are provided in Table 1. Iterative analysis determined seven common themes by which studies investigating different applications could be grouped and compared. A more detailed analysis is subsequently provided according to the specific IRT application.

**Table 1:** Characteristics of the included studies grouped by location.

First Author, year of publication (reference)	Study design	Sample size (*n*)	Variables measured using IRT	Study description
Adult emergency department				
Katz, 2008 (11)	Observational (case–control)	164	∆T _(thigh to foot)_	Comparison of ∆T in compartment syndrome and control legs
Sillero-Quintana, 2015 (12)	Observational	201	∆T _(affected and unaffected limbs)_	Quantification of ∆T in injured limbs
Ko, 2017 (13)	Prospective observational (cohort)	72	∆T _(affected and unaffected skin)_	Association of ∆T with clinician diagnosed cellulitis
Coats, 2018 (14)	Observational (case–control)	56	∆T _(upper arm to finger)_	Comparison of ∆T between healthy, fever and sepsis patient groups
Holm, 2018 (15)	Prospective observational (cohort)	198	∆T _(inner canthus to earlobe, nose tip or finger)_	Association of ∆T with all-cause 30-day mortality
Zhang, 2021 (16)	Observational	154	Temperature of skin overlying the nasopharynx	Quantification of nasopharyngeal skin temperature in patients presenting with upper respiratory tract infection
Brzezinski, 2021 (17)	Observational (case–control)	101	Temperature distribution on posterior thorax thermograph	Comparison of temperature distribution in COVID-19 and control patients
Hejgaard Jensen, 2021 (18)	Prospective observational (cohort)	726	∆T _(inner canthus to earlobe, nose tip or finger)_	Association of ∆T with all-cause 30-day mortality
der Strasse, 2022 (19)	Observational	45	∆T _(affected and unaffected limbs)_	Quantification of ∆T in bone fractures
Aldred, 2023 (20)	Prospective observational	136	Air temperature fluctuations at the nostrils	Accuracy in respiratory rate estimation compared with manual counting
Adult intensive care unit				
Ferraris, 2018 (21)	Prospective observational	46	Cutaneous temperature at the knee	Association of knee temperature with mottling score in patients with septic shock
Mayrovitz, 2018 (22)	Prospective observational	100	∆T _(sacrum and reference area)_	Association between a diagnosis of cardiovascular disease in patients with high and low risk of pressure injury as categorised by ∆T
Koerner, 2019 (23)	Interventional	114	∆T _(heels, sacrum or coccyx and reference area)_	Rates of hospital acquired pressure injury compared to historical values following the introduction of an IRT pressure injury assessment
Doesburg, 2019 (24)	Prospective observational	128	∆T _(cannula insertion and reference sites)_	Association of ∆T with Visual Infusion Phlebitis score
Amson, 2020 (25)	Prospective observational	61	∆T _(urinary or tympanic measured core temperature and IRT measured forearm, index finger, knee and hallux)_	Association of ∆T with 8-day mortality in patients with sepsis
Chan, 2020 (26)	Prospective observational	27	Air temperature fluctuations at the nostrils	Accuracy for respiratory rate estimation compared with manual counting and thoracic impedance
Luo, 2022 (27)	Prospective cohort	373	Temperature inhomogeneity on lower limb thermographs	Association of thermal inhomogeneity and in-hospital mortality after major cardiac surgery
Kazune, 2023 (28)	Prospective observational	81	∆T _(hotspot and adjacent skin on thermograph of anterior thigh)_	Comparison of ∆T between groups of patients with different mottling scores in the context of septic shock
Gutowski, 2024 (29)	Prospective cohort	116	Mean temperature of the distal phalanges	Correlation between perfusion parameters (IRT measured, renal cortex perfusion, peripheral oxygen saturation and capillary refill time) in patients with severe COVID-19
Hasanin, 2024 (30)	Prospective observational	56	∆T _(inner canthus to great toe)_	Association of ∆T with in-hospital mortality in patients with septic shock
Paediatric emergency department				
Saxena, 2008 (31)	Case series	285	Measured variables depended on surgical condition	Descriptive study of IRT applications for various acute paediatric surgical conditions
Silva, 2012 (32)	Observational	51	Location of maximal skin temperature in injured limb	Correlation between maximal skin temperature location and site of pain or fracture
Sanchis Sanchez, 2015 (33)	Observational	133	∆T _(injured and non-injured limbs)_	Accuracy of using IRT measured values for the diagnosis of fracture
Owen, 2018 (34)	Observational (case–control)	30	∆T _(affected and unaffected limb)_	Comparison of ∆T in clinically diagnosed soft tissue injury, fracture and irritable hip groups of patients who presented with acute limp
Reed, 2020 (35)	Observational (case–control)	40	∆T _(injured and non-injured limb)_	Comparison of ∆T in fractured and sprained groups of patients who presented with wrist injury
Paediatric intensive care unit				
Nagori, 2019 (36)	Prospective longitudinal	51	∆T _(abdomen to foot)_	Correlation of ∆T with binary shock index (incorporating median heart rate and arterial systolic pressure)
Shcherbakova, 2022 (37)	Prospective observational cohort	36	∆T _(inner canthus to finger)_	Correlation of ∆T with changes in cardiac output assessed clinically
Vats, 2022 (38)	Observational (case–control)	22	∆T _(abdomen to foot)_	Correlation of ∆T with binary shock index (incorporating median heart rate and arterial systolic pressure)
Bridier, 2023 (39)	Prospective observational	41	∆T _(inner canthus to hallux)_	Correlation of ∆T with oxygen extraction ratio in patients following cardiac surgery
Neonatal intensive care unit				
Barcat, 2017 (40)	Observational	29	∆T _(abdomen to pectoral region, thigh, hand, foot or eye)_	Variation of ∆T values with wakefulness episodes

∆T, temperature difference.

### 3.1 Quality appraisal

**Figure 2:**
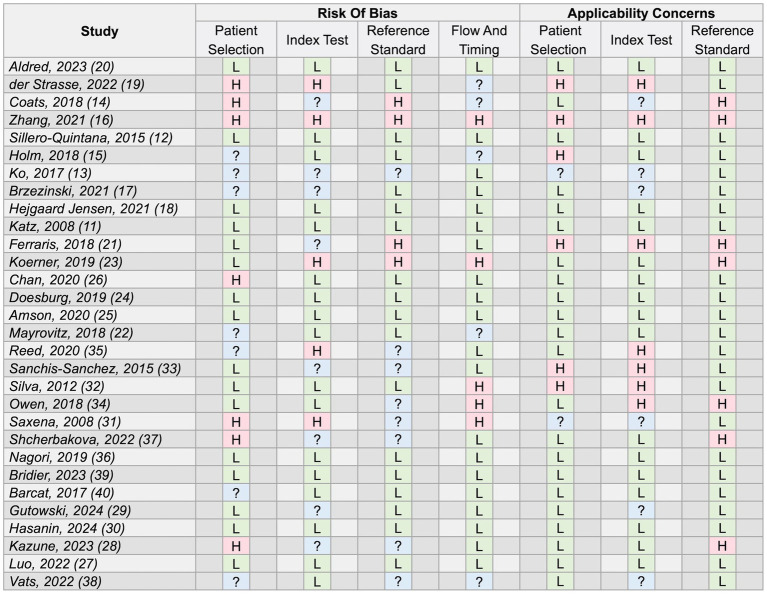
Full results of study quality appraisal using the Quality Assessment tool for Diagnostic Accuracy Studies 2 (QUADAS-2) tool (9). L, low risk/concerns; H, high risk/concerns; ?, unclear risk/concerns for the respective domains.

The proportion of studies with a low risk of bias rating in the patient selection, index test, reference standard and timing domains was 53, 57, 60 and 67%, respectively. The proportion of studies with a low-risk rating for concerns around the applicability of their findings in the patient selection, index test and reference standard domains were 73, 57 and 77%, respectively. Ten studies scored low risk ratings across all domains (11, 12, 18, 20, 24, 25, 27, 30, 36, 39). One scored high-risk ratings across all domains (16). Overall, 17% of domain assessments were given an “unclear” rating due to insufficient information provided by study authors. A full breakdown of the quality appraisal assessment is provided in Figure 2.

### 3.2 Analysis by theme

#### 3.2.1 Application purpose

IRT was most frequently used for diagnosis (*n* = 18). Observations made using IRT were also related to mortality risk or disease severity in certain conditions, specifically shock and COVID-19. Two studies investigated IRT for monitoring of respiratory rate (20, 26). Other studies made observations using IRT sometimes of novel phenomena (40).

#### 3.2.2 Mechanism underlying application

There were two predominant mechanisms that underpinned the application of IRT in several of the studies. The first mechanism describes temperature changes associated with a localised inflammatory response. Infection or injury causes inflammatory mediator release resulting in localised vasodilation. Increased blood flow to an area raises the skin temperature which may then be visualised using IRT. The second mechanism describes temperature changes associated with microvascular dysfunction at the skin secondary to a systemic insult. Shock describes the inability of the cardiovascular system to maintain sufficient oxygen delivery to the tissues. It may be precipitated by severe infection (sepsis) amongst other causes. In shock, the body may restrict blood supply to peripheral tissues to maintain perfusion to the vital central organs. Peripheral vasoconstriction causing a reduced blood supply distally may be reflected by a cutaneous temperature gradient which may be visualised using IRT.

#### 3.2.3 Location

**Figure 3:**
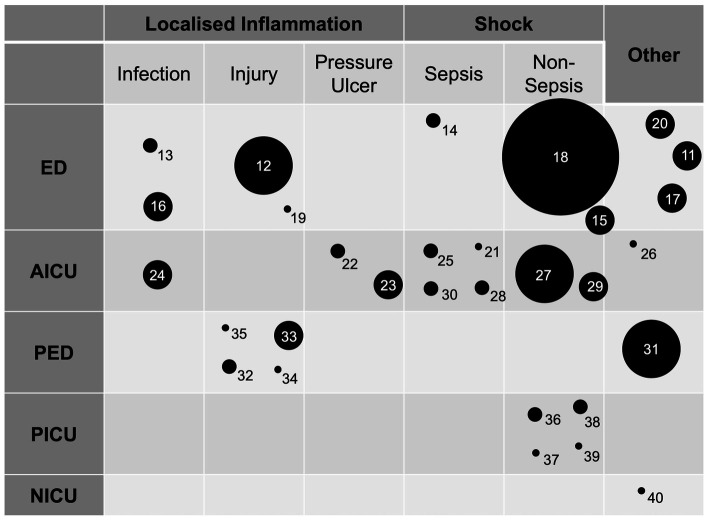
A plot of the included studies arranged by location and application. Circle diameter is proportional to number of study participants arranged into five groups (smallest to largest circle representing 0–25, 26–50, 51–100, 101–200, 201–400, 401–800 participants respectively). Numbers identify individual studies and correspond to the reference list. ED, emergency department; AICU, adult intensive care unit; PED, paediatric emergency department (includes single paediatric surgical assessment unit study); PICU; paediatric intensive care unit; NICU, neonatal intensive care unit.

In adult patients, ten (33%) studies were performed in emergency departments and ten (33%) in intensive care units. In paediatric patients, four (13%) studies were conducted in the emergency department and four (13%) in the intensive care unit. One study was performed in the neonatal intensive care unit and one in a paediatric acute surgical assessment clinic. There were no included studies that were performed in the prehospital setting or acute medical unit. The distribution of studies according to location is summarised in Table 1 and Figure 3.

#### 3.2.4 Stage of development

The majority of authors identified their studies as exploratory or assessing feasibility (*n* = 16) (11, 12, 14–16, 19, 21, 22, 26, 27, 31, 32, 34, 35, 37, 39). Few were described as validation or diagnostic accuracy studies (*n* = 8) (13, 17, 18, 20, 24, 33, 36, 38).

#### 3.2.5 Advantages of infrared thermography

Across the studies there were common features of IRT that made it an attractive and novel tool to study. The ability to image temperature gradients was advantageous for investigating microcirculatory dysfunction. Quantifying surface temperatures was beneficial when assessing injury or localised infections. Some studies found IRT as a non-ionising form of imaging beneficial, particularly those investigating musculoskeletal injuries in paediatric patients (32–35). The use of a non-contact, continuous imaging modality was beneficial to studies applying IRT for monitoring purposes (20, 26, 36–38).

#### 3.2.6 Limitations of infrared thermography

Studies did not often discuss the limitations of IRT in their reports. Of the studies that did, five described the need for an acclimatisation period (20, 22, 32, 33, 35). Two described the requirement of a standardised environment as potentially limiting the applicability of IRT to clinical practise (14, 18). The need to remove skin coverings to enable the device to be used was frequently mentioned (11, 19, 20, 31–33, 35, 38). Anti-inflammatory use, peripheral vascular disease and ice pack application were identified as potential confounders that excluded some recruits from study participation. Two studies acknowledged limitations cause by inadequate resolution of the IRT device used (37, 38).

#### 3.2.7 Measured variables

There were commonly measured variables across the group of studies (Table 1). In studies investigating conditions affecting one side of the body (e.g., limb injury) a common comparison was the affected with the unaffected side, from which a temperature difference could be obtained (11–13, 19, 33–35). Alternatively, affected and unaffected areas within the same limb could also be compared (13, 24, 35). Broadly, temperature difference (i.e., between affected and unaffected areas) tended to be a more valuable measure than comparing absolute temperature values. The use of an internal control enables individual basal temperature variations to be accounted for. In studies investigating core to peripheral temperature gradients, commonly the central temperature measurement used was taken from the internal canthus (15, 18, 30, 37, 39). This is based on international guidance (2). Peripheral measurements were most commonly obtained from the finger, nose or toe (14, 15, 18, 25, 29, 30, 36–39).

### 3.3 Analysis by application

In addition to common mechanisms, there was overlap in the individual conditions in which IRT was studied across the different departments. Studies that used IRT to study localised inflammation investigated cellulitis (13), pressure ulcers (22, 23), and limb injury (*n* = 5) (12, 19, 32, 33, 35). Studies which used IRT to study core to peripheral temperature gradients commonly studied septic shock (*n* = 5) (14, 21, 25, 28, 30). The applications are discussed in further detail in the following section and also summarised in Figure 3.

#### 3.3.1 Localised inflammation

##### 3.3.1.1 Infection

Three studies used IRT to identify raised skin temperatures from inflammation secondary to localised infections (13, 16, 24). One study applied IRT for the diagnosis of cellulitis in adults in the emergency department (13). They found significantly different maximum temperatures and a significant temperature difference between affected and unaffected limbs. They further developed and validated a diagnostic model incorporating an IRT measured temperature difference of ≥0.47°C to achieve a Positive Predictive Value (PPV) of 85.7% and a Negative Predictive Value (NPV) of 100% for the diagnosis of cellulitis (13). One study investigated localised temperature changes around the nasopharynx in adult patients presenting to the emergency department with upper respiratory tract infection symptoms (16). Patients with tympanic temperatures ≥37.1°C also had higher absolute cutaneous temperatures overlying the nasopharynx. This study did not correlate findings with an objective upper respiratory tract infection diagnosis making it difficult to interpret the usefulness of this measure. One study applied IRT for the early objective identification of phlebitis in adult ICU patients (24). Localised inflammation, and therefore raised skin temperature, may occur physiologically in response to infection at the insertion site of a cannula. This study demonstrated significantly higher absolute temperatures and greater temperate differences (with reference sites) at cannula insertion sites with any visual signs of phlebitis (visual infusion phlebitis score ≥ 1) compared to those without (visual infusion phlebitis score = 0). This group presented consistent findings from a pilot and larger validation study.

##### 3.3.1.2 Limb injuries

Six studies investigated IRT as a diagnostic adjunct for limb injury presentations to the emergency department (12, 19, 32–35). Four studies involved paediatric patients (32–35). One of these used IRT as an examination adjunct for presentations of acute limp in children (34). Inflammation secondary to injury or infection, causing a raised skin temperature over the affected region, is the biological mechanism underpinning the use of IRT in these conditions. IRT was selected for use in these patients because of its non-ionising properties. Broadly, these studies aim to use IRT as a screening tool or examination adjunct to identify patients who may benefit from further diagnostic imaging. The earliest paediatric limb injury study initially found a poor correlation between maximum cutaneous temperature of the affected limb and fracture site (32). However, two subsequent studies measured temperature differences between affected and unaffected limbs with greater success (33, 35). One incorporated limb temperature difference into a decision tree model which demonstrated an Area Under the receiver operating characteristic Curve (AUC) of 0.97 (33). The single acute limp study demonstrated higher median temperatures in affected limbs when any pathology was present, as identified from medical records (34). Two studies were conducted with adult patients (12, 19). Both demonstrated feasibility, with a statistically significant difference in temperature between affected and unaffected limbs. The adult studies are at an earlier stage of development compared to the paediatric limb injury studies where diagnostic accuracy has been investigated (33).

##### 3.3.1.3 Pressure ulcers

Two studies applied IRT in the adult ICU for the prevention of Hospital Acquired Pressure Ulcers (HAPUs) (22, 23). The studies present conflicting mechanisms for the thermal changes associated with HAPU formation. One argued that reduced blood flow, for example in patients with peripheral vascular disease, results in cooler peripheral temperatures and promotes the risk of HAPU formation (22). The study proposing this mechanism did not produce findings to support this. A temperature difference between pressure and reference areas of >1.5°C was not associated with risk factors for impaired perfusion or HAPU formation. The subsequent study proposed that inflammation in the early stages of HAPU formation rases skin temperature (23). This study used IRT to detect thermal differences between reference and pressure areas (raised or lowered temperatures) and classified those patients as at risk of HAPU. HAPU prevention interventions were then applied to this group. Methodological aspects of this study limit any findings. Confounders were not controlled for between the intervention groups. Additionally, the outcome measure compared HAPU incidence to an historic rate in that setting, without adjustment for confounding characteristics. There is also a potential conflict of interest arising from the study funding.

#### 3.3.2 Monitoring

Two studies applied IRT for non-contact respiratory rate monitoring albeit in different locations (20, 26). IRT was used to continuously monitor thermal air changes at the nares to quantify respiratory rate. Expired gases are warmed by the body and respiratory tract and can therefore be visualised by IRT when exhaled. In non-intubated adult patients in ICU, IRT demonstrated greater accuracy and less bias than thoracic impedance for measuring respiratory rate when compared to manual estimations (26). In adults in the emergency department, IRT demonstrated an error rate of ±4 breaths per minute when compared with manual respiratory rate estimations (20).

#### 3.3.3 Microcirculatory dysfunction

Thirteen studies used IRT to assess microcirculatory dysfunction. In severe illness or septic shock, the sympathetic nervous system causes constriction of dermal arterioles to divert blood towards vital organs. Reduced cutaneous blood flow reduces peripheral skin temperature and may cause mottling. Core body temperature is usually maintained producing a measurable Central to Peripheral Temperature Difference (CPTD).

##### 3.3.3.1 Adult septic shock

In adult patients, five studies investigated IRT applications in septic shock (14, 21, 25, 28, 30). Three studied CPTDs with IRT. Feasibility was demonstrated when a significant CPTD (upper arm to fingers) present in healthy patients was absent in pyrexial patients in the emergency department (14). The sepsis group in this study was too small (5 patients) to draw meaningful conclusions (14). Further work in the ICU found that a core (tympanic or urinary) to index finger (measured using IRT) temperature gradient of >7°C was associated with 8-day mortality (including after adjustment for individual Sequential Organ Failure Assessment scores) in patients with suspected sepsis requiring vasopressors (25). The most recent of these studies similarly found that non-survivors of a post-operative ICU admission with sepsis had significantly greater CPTDs (canthus to toe) than survivors. However, they also demonstrated that this measure had no significant accuracy in predicting in-hospital mortality in this group whereas other measures such as capillary refill time or serum lactate did (30).

The two other adult sepsis studies proposed that a reduction in cutaneous blood flow, often visible clinically as mottling, could be objectively measured using IRT as a reduction in cutaneous temperature (21, 28). However, both studies showed that neither cutaneous temperature (measured with IRT at the knee) nor mottling score were associated with 28-day mortality (21, 28).

##### 3.3.3.2 Adult shock

Two studies investigated CPTD measured with IRT and mortality in a broader group of medical patients in the emergency department (15, 18). Both found a significant association between CPTD (inner canthus and nose) and 30-day mortality. The larger later study found CPTD to be comparable to National Early Warning Score, clinical intuition, and age as predictors of mortality (18). There are some potential reasons for the different findings investigating mortality and CPTD. Studies used different end points (30 vs. 90-day mortality) and different patients groups. Additionally, some authors acknowledge that variation in measurement conditions (ambient temperature) may have affected their findings (18).

One study investigated five novel variables to quantify thermal inhomogeneity from IRT images of the anterior thigh in patients following major cardiac surgery with risk factors for hypoperfusion (27). Two measurements (standard deviation and low-temperature-area-rate) had a similar accuracy to capillary refill time in predicating in-hospital mortality. The accuracy of the models improved when multiple variables were combined together or with conventional measures of hypoperfusion (27). The final adult shock study investigated an association between COVID-19 disease severity and markers of microcirculatory dysfunction, one of which was mean fingertip temperature measured with IRT. The correlation between fingertip temperature and COVID-19 disease severity only tended towards significance (*p* = 0.054) (29).

##### 3.3.3.3 Paediatric shock

Four studies investigated CPTDs and shock prediction in the paediatric ICU (36–39). The feasibility of using IRT for measuring CPTD (epicanthus to finger) in paediatric patients was demonstrated by one small study (37). They did not find any relationship between CPTD and participant characteristics (pathology, comorbidities, vital signs). Another investigated CPTD (epicanthus to toe) in paediatric patients following cardiac surgery (39). No correlation was found between CPTD and oxygen extraction coefficient (a marker for shock). Two studies from the same group are probably the most significant across both adults and children investigating IRT use in shock (36, 38). The earlier study developed and validated a machine learning algorithm to detect and predict shock from thermal images in critically ill children. The investigators taught an algorithm to identify regions of interest in IRT images (foot and abdomen) from which CPTDs could be calculated automatically. In combination with pulse rate, automatically calculated CPTDs had a 73% accuracy at detecting and predicting shock at 0 and 3 h (AUC 0.75, 0.77 respectively). Subsequently, a similar methodology was applied to IRT videos which improved the accuracy of the model further with the best performance shown at 5 h (AUC 0.81) (38).

#### 3.3.4 Other applications

One study used machine learning and an image processing algorithm to diagnose COVID-19 from IRT images of the posterior thorax in adult patients in the emergency department (17). Two image processing parameters were specifically identified then validated in a model which demonstrated an AUC of 0.85 and sensitivity of 92%. The biological mechanism underpinning this application is not fully understood, as acknowledged by the study authors. They speculated that inflammation and microvascular dysfunction in lungs were reflected by thermal changes across the skin of the thorax.

Another study conducted in the adult emergency department found a significant association between the development of compartment syndrome and a greater thigh-foot temperature gradient as measured using IRT within 4 h of injury (11). The proposed underlying mechanism suggested that compression of blood vessels, from raised compartment pressures, reduces the circulation of warm blood through the compartment thus reducing its temperature. This small feasibility study was limited by a retrospective and subjective reference diagnostic standard (medical records) as opposed to a contemporaneous objective test (compartment pressures) for the diagnosis of compartment syndrome.

The systematic review process identified a descriptive study of IRT use in acute paediatric surgical presentations (31). This study described the potential use of IRT in diagnosing and assessing treatment response across a range of several presentations, including haemangiomas, vascular malformations, varicoceles, acute thrombosis and digit reimplantation following amputation. The study was conducted as a large case series of IRT applications across 10 years therefore only descriptive results are presented without reference to case selection methodology.

A single study was conducted in the neonatal intensive care unit (40). This was an exploratory study to determine if peripheral vasodilatation promoted sleep onset in preterm neonates (a phenomenon that is observed in adults). The study measured cutaneous temperature at 6 sites using IRT. The mechanism outlined by the study suggested that vasodilatation would result in more peripheral blood flow and therefore more homogenous cutaneous temperatures or a narrower CPTD. The investigators found shorter wakefulness episodes (measured with polysomnography) to be significantly associated more homogenous cutaneous temperatures and narrower CPTDs (indicating vasodilation) at the end of a wakefulness period (suggesting sleep onset). This was a novel observation in the preterm neonatal population, made using IRT.

## 4 Discussion

These data provide a descriptive summary of the potential applications of IRT in the care of acutely unwell patients. Studies were performed in emergency departments and intensive care units, but not prehospital or acute medical unit settings, and involved adult, paediatric and neonatal patients. We have identified a range of situations where IRT has been applied for diagnosis, risk prediction or monitoring. The applications of IRT in acute care are broad but there are few studies that consistently investigate the same conditions or outcomes. IRT is a relatively novel technology, which may explain why the majority of included studies aimed to assess feasibility as opposed to diagnostic accuracy. We have outlined the significant potential for IRT to be a valuable clinical tool in the management of acutely unwell patients however, further investigation is required to establish use clinically.

Through our analysis, we have identified two predominant mechanisms that underpin the IRT technology in acute care. Within these broad categories, we have also identified clusters of studies relating to specific applications. This has subsequently informed our recommendations for further research. Firstly, skin temperature changes associated with localised inflammation may be imaged using IRT for the diagnosis of cellulitis, phlebitis associated with vascular access and paediatric limb injuries. We recommend that large, multicentre, high quality, diagnostic accuracy studies, that compare IRT with current ‘gold standard’ investigations, would be appropriate to further develop IRT for use in the three conditions listed.

Secondly, core-to-peripheral temperature differences may be estimated for the early identification or mortality risk prediction of shock. In adult patients, there is a lack of consistency in the evidence for this application. Across the studies, different variables (CPTD vs. thermal distributions) were measured using IRT from different anatomical sites in different patient groups (medical, septic and surgical). A range of different outcome measures have also been studied. Earlier mortality endpoints plausibly appear to have stronger associations with the IRT measured variables for shock. However, these variables have yet to be successfully incorporated into accurate predictive models.

In paediatric patients, the evidence is stronger for IRT use in shock. The most significant investigations successfully use IRT images or videos and a machine learning algorithm for the early identification of shock in paediatric ICU patients (36, 38). This work demonstrates the substantial impact that IRT could have clinically in particular when combined with emerging computing strategies such as artificial intelligence for image processing. We identified three studies that combined machine learning and IRT (17, 36, 38). All used machine learning to automate image processing. This expedites an otherwise time-consuming process, which is particularly valuable in urgent medical conditions such as sepsis that require immediate management.

Further research could focus on replicating the paediatric studies in multiple centres, adult patients or different departments. The physiological differences in thermoregulation between children and adults should be considered. In paediatric patients, immaturity of the thermoregulatory system may cause variable physiological responses. There are few published studies on IRT in children. Until more data are available to assist interpretation, IRT should be used with caution for diagnosis and monitoring in children. Future researchers may wish to consider whether IRT best enables the early diagnosis of shock as opposed to estimating mortality risk. Establishing consistently used outcome measures across studies in this area would additionally strengthen the future evidence base.

To our knowledge, this is the first systematic review of the applications of IRT specific to the acute care setting. We provide a descriptive and critical overview of the current literature. We aim to guide future researchers towards the current evidence gaps that require addressing before IRT may be applied in clinical practise.

### 4.1 Strengths and limitations

The key strengths of our work include the systematic search strategy used and the rigorous application of transparent review methodology. We address a novel review topic aiming to provide a useful summary of an expanding area of research. We searched a range of sources including grey literature in order to minimise publication bias and capture the breadth of available literature. The main aim of this study was to describe and outline current evidence therefore our search strategies and inclusion criteria were intentionally broad. We defined acute care by location so as to capture studies performed on patients in these clinical environments and to avoid inappropriately limiting findings by searching only for certain conditions.

However, this did result in a heterogenous group of included studies meaning that quantitative analysis was not appropriate. Given the novelty of IRT technology in acute care, many studies were early feasibility studies involving small patient numbers with inconsistent reference and outcome measures. Whilst the quality of some studies was high, many others were prone to bias which overall limits the strength of evidence presented in this review. In addition, we selected a quality assurance tool based on the most common purpose (diagnostic) of IRT revealed by our scoping review. However, the heterogeneity of study types we identified means that our quality assurance tool is unlikely to have been applicable in all cases. Lastly, we identified acute care by location as opposed to pathology. It is possible that studies with conditions relevant to acute care may have been conducted outside these departments and so would not have been captured by our searches.

## 5 Conclusion

This review confirms that IRT is a potentially useful tool in the care of acutely unwell patients. The potential is greater still when combined with wider advances such as machine learning technology. However, further high-quality validation studies are needed for IRT to become a realistic adjunct to clinical practise. We recommend the applications of IRT in acute care to be an important and potentially fruitful area of further research.

## Data availability statement

The original contributions presented in the study are included in the article/Supplementary material, further inquiries can be directed to the corresponding author.

## Author contributions

SS: Conceptualization, Formal analysis, Investigation, Methodology, Project administration, Writing – original draft. PD: Investigation, Methodology, Resources, Writing – review & editing. JT: Supervision, Writing – review & editing. MC: Conceptualization, Formal analysis, Supervision, Writing – review & editing.
