# Supplementary material for: Uses of infrared thermography in acute illness: a systematic review
Source: Front Med (Lausanne). 2024 Jun 24;11:1412854. doi: 10.3389/fmed.2024.1412854 (PMC11232369; doi:10.3389/fmed.2024.1412854)
Supplement: Supplementary file 1 [file Table_1.DOCX]

**Supplementary Material**

The exact search strategies are provided that were used to identify studies for systematic review.

**1 Embase and MEDLINE**

Embase <1974 to 2024 March 27>, Ovid MEDLINE(R) ALL <1946 to March 27, 2024>

1 Thermography/

2 thermograph*.mp.

3 (temperature adj2 mapping*).mp. [mp=ti, ab, hw, tn, ot, dm, mf, dv, kf, fx, dq, nm, ox, px, rx, ui, sy]

4 (infrared adj2 (imag* or camera*)).mp. [mp=ti, ab, hw, tn, ot, dm, mf, dv, kf, fx, dq, nm, ox, px, rx, ui, sy]

5 (thermal adj2 (imag* or camera*)).mp. [mp=ti, ab, hw, tn, ot, dm, mf, dv, kf, fx, dq, nm, ox, px, rx, ui, sy]

6 infrared rays/

7 infrared ray*1.mp.

8 "heat wave*1".mp. [mp=ti, ab, hw, tn, ot, dm, mf, dv, kf, fx, dq, nm, ox, px, rx, ui, s

9 or/1-8

10 (ITU or ICU or (intensive adj2 (care or treatment))).mp. or INTENSIVE CARE/ or INTENSIVE CARE UNITS/ or exp CRITICAL CARE/ or (critical adj2 care).mp.

11 emergency department.mp. or Emergency Service, Hospital/

12 Intensive Care Units, Neonatal/ or scbu.mp.

13 Intensive Care, Neonatal/ or NICU.mp.

14 aicu.mp.

15 Intensive Care Units, Pediatric/ or PICU.mp.

16 Critical Illness/

17 (hdu or "high dependency").mp. [mp=ti, ab, hw, tn, ot, dm, mf, dv, kf, fx, dq, nm, ox, px, rx, ui, sy]

18 Emergency Service, Hospital/ or Emergency Medical Services/ or "accident and emergency".mp.

19 Extracorporeal Membrane Oxygenation/ or ecmo.mp.

20 (AMU or MAU or "acute medical").mp. [mp=ti, ab, hw, tn, ot, dm, mf, dv, kf, fx, dq, nm, ox, px, rx, ui, sy]

21 Emergency Medicine/

22 (prehospital* or pre-hospital* or pre hospital* or ambulance* or paramedic* or EMS).mp. [mp=ti, ab, hw, tn, ot, dm, mf, dv, kf, fx, dq, nm, ox, px, rx, ui, sy]

23 PHEM.mp.

24 Emergency Responders/

25 "out of hospital".mp.

26 ("emergency room" or "emergency department").mp. [mp=ti, ab, hw, tn, ot, dm, mf, dv, kf, fx, dq, nm, ox, px, rx, ui, sy]

27 or/10-26

28 9 and 27

29 thermography/

30 thermograph*.mp.

31 (temperature adj2 mapping*).mp. [mp=ti, ab, hw, tn, ot, dm, mf, dv, kf, fx, dq, nm, ox, px, rx, ui, sy]

32 (infrared adj2 (imag* or camera*)).mp. [mp=ti, ab, hw, tn, ot, dm, mf, dv, kf, fx, dq, nm, ox, px, rx, ui, sy]

33 infrared camera/

34 (thermal adj2 (imag* or camera*)).mp. [mp=ti, ab, hw, tn, ot, dm, mf, dv, kf, fx, dq, nm, ox, px, rx, ui, sy]

35 infrared radiation/

36 infrared ray*1.mp.

37 "heat wave*1".mp.

38 (ITU or ICU or (intensive adj2 (care or treatment)) or (critical adj2 care)).mp.

39 intensive care/

40 exp intensive care unit/

41 emergency department.mp. or emergency ward/

42 scbu.mp.

43 newborn intensive care/ or neonatal intensive care unit/ or NICU.mp.

44 aicu.mp.

45 pediatric intensive care unit/ or picu.mp.

46 critical illness/

47 high dependency unit/

48 (hdu or "high dependency").mp. [mp=ti, ab, hw, tn, ot, dm, mf, dv, kf, fx, dq, nm, ox, px, rx, ui, sy]

49 "accident and emergency".mp. or emergency health service/

50 veno-venous ECMO/ or veno-arterial ECMO/ or ecmo.mp.

51 (AMU or MAU or "acute medical").mp.

52 emergency medicine/

53 (prehospital* or pre-hospital* or pre hospital* or ambulance* or paramedic* or EMS).mp.

54 PHEM.mp.

55 rescue personnel/

56 "out of hospital".mp.

57 ("emergency room" or "emergency department").mp.

58 or/29-37

59 or/38-57

60 58 and 59

**2 CINAHL**

# Query

S1 (MH "Thermography")

S2 thermograph*

S3 temperature n2 mapping*

S4 infrared n2 (imag* or camera*)

S5 thermal n2 (imag* or camera*)

S6 infrared ray*

S7 "heat wave*"

S8 S1 OR S2 OR S3 OR S4 OR S5 OR S6 OR S7

S9 ITU or ICU or (intensive n2 (care or treatment))

S10 (MH "Intensive Care Units") OR (MH "Intensive Care Units, Pediatric+")

S11 (MH "Critical Care")

S12 critical n2 care

S13 (MH "Emergency Service") OR "emergency department"

S14 (MH "Pediatric Critical Care Nursing") OR (MH "Neonatal Intensive Care Nursing") OR "scbu or NICU or PICU"

S15 aicu

S16 (MH "Critical Illness")

S17 hdu or "high dependency"

S18 ""accident and emergency""

S19 (MH "Emergency Medical Services")

S20 (MH "Extracorporeal Membrane Oxygenation") OR "ecmo"

S21 AMU or MAU or "acute medical"

S22 prehospital* or pre-hospital* or pre hospital* or ambulance* or paramedic* or EMS or PHEM

S23 (MH "Emergency Medical Technicians")

S24 (MH "Prehospital Care")

S25 "out of hospital"

S26 ("emergency room" or "emergency department")

S27 S9 OR S10 OR S11 OR S12 OR S13 OR S14 OR S15 OR S16 OR S17 OR S18 OR S19 OR S20 OR S21 OR S22 OR S23 OR S24 OR S25 OR S26

S28 S8 AND S27

**3 Cochrane CENTRAL**

ID Search

#1 MeSH descriptor: [Thermography] explode all trees

#2 thermograph*

#3 temperature near/2 mapping*

#4 infrared near/2 (imag* or camera*)

#5 thermal near/2 (imag* or camera*)

#6 MeSH descriptor: [Infrared Rays] explode all trees

#7 infrared ray*

#8 heat wave*

#9 {or #1-#8}

#10 ITU or ICU or (intensive near/2 (care or treatment))

#11 MeSH descriptor: [Intensive Care Units] explode all trees

#12 MeSH descriptor: [Critical Care] explode all trees

#13 MeSH descriptor: [Intensive Care, Neonatal] explode all trees

#14 MeSH descriptor: [Intensive Care Units, Pediatric] explode all trees

#15 critical near/2 care

#16 MeSH descriptor: [Emergency Service, Hospital] explode all trees

#17 emergency department

#18 SCBU or NICU or PICU or AICU

#19 MeSH descriptor: [Critical Illness] explode all trees

#20 hdu or "high dependency"

#21 MeSH descriptor: [Emergency Medical Services] explode all trees

#22 "accident and emergency"

#23 MeSH descriptor: [Extracorporeal Membrane Oxygenation] explode all trees

#24 ecmo

#25 AMU or MAU or "acute medical"

#26 MeSH descriptor: [Emergency Medicine] explode all trees

#27 prehospital* or pre-hospital* or pre hospital* or ambulance* or paramedic* or EMS or PHEM

#28 MeSH descriptor: [Emergency Responders] explode all trees

#29 "out of hospital"

#30 ("emergency room" or "emergency department")

#31 {or #10-#30}

#32 #9 and #31

**4 MedRxiv**

www.medrxiv.org

"thermography critical care"

“thermography intensive care”

**5 ISRCTN**

https://www.isrctn.com/search

Thermography

Infrared camera
